# Biological Characteristics of Severe Combined Immunodeficient Mice Produced by CRISPR/Cas9-Mediated *Rag2* and *IL2rg* Mutation

**DOI:** 10.3389/fgene.2019.00401

**Published:** 2019-04-30

**Authors:** Yong Zhao, Peijuan Liu, Zhiqian Xin, Changhong Shi, Yinlan Bai, Xiuxuan Sun, Ya Zhao, Xiaoya Wang, Li Liu, Xuan Zhao, Zhinan Chen, Hai Zhang

**Affiliations:** ^1^Laboratory Animal Center, Air Force Medical University, Xi’an, China; ^2^Department of Microbiology, Air Force Medical University, Xi’an, China; ^3^Department of Cell Biology, National Translational Science Center for Molecular Medicine, Air Force Medical University, Xi’an, China; ^4^College of Veterinary Medicine, Northwest A&F University, Yangling, China; ^5^Key Laboratory for Space Bioscience and Biotechnology, School of Life Sciences, Northwestern Polytechnical University, Xi’an, China; ^6^National Translational Science Center for Molecular Medicine, Air Force Medical University, Xi’an, China

**Keywords:** *Rag2*, *IL2rg*, CRISPR/Cas9, severe combined immunodeficient mice, biological characteristic

## Abstract

Clustered regularly interspaced short palindromic repeats (CRISPR)/CRISPR-associated (Cas)9 is a novel and convenient gene editing system that can be used to construct genetically modified animals. Recombination activating gene 2 (*Rag2*) is a core component that is involved in the initiation of V(D)J recombination during T- and B-cells maturation. Separately, the interleukin-2 receptor gamma chain gene (*IL2rg*) encoded the protein-regulated activity of natural killer (NK) cells and shared common receptors of some cytokines. *Rag2* and *IL2rg* mutations cause immune system disorders associated with T-, B-, and NK cell function and some cytokine activities. In the present study, 2 single-guide RNAs (sgRNAs) targeted on *Rag2* and *IL2rg* genes were microinjected into the zygotes of BALB/c mice with Cas9 messenger RNA (mRNA) to create *Rag2*/*IL2rg*^-/-^ double knockout mice, and the biological characteristics of the mutated mice were subsequently analyzed. The results showed that CRISPR/Cas9-induced indel mutation displaced the frameshift of *Rag2* and *IL2rg* genes, resulting in a decrease in the number of T-, B-, and NK cells and the destruction of immune-related tissues like the thymus and spleen. *Mycobacterium tuberculosis* 85B antigen could not induce cellular and humoral immune response in mice. However, this aberrant immune activity compromised the growth of several tumor heterogenous grafts in the mutated mice, including orthotopic and subcutaneous transplantation tumors. Thus, *Rag2/IL2rg*^-/-^ knockout mice possessed features of severe combined immunodeficiency (SCID), which is an ideal model for human xenograft.

## Introduction

The construction of chimeras of a rodent animal model that harbors human tissues has provided valuable *in vivo* assay systems in biomedical research. To do this, aberrant immune-related genes make it possible to construct chimeric rodent animals. The nude mouse (or athymic nude mouse) was first described by Flanagan (1966), which involved a spontaneous mutation in the *Foxn1* gene, resulting in a lack of fur development and impaired T-cell function (Schorpp et al., 1997). Thereafter, CBA/N and Beige mice, which boasted mutations in the *xid* and *beige* genes, respectively, leading to B-cell- and natural killer (NK)-cell-mediated immune-response failure, were also discovered (Clark et al., 1981; Klaus et al., 1997). After that, *prkdc* gene and *Rag2* mutation mouse, which showed T- and B-cell dysregulation, were defined as a severe combined immunodeficiency (SCID) mouse and used widely in biomedical research (Shinkai et al., 1992; Greiner et al., 1998). Subsequently, SCID mice were greatly improved by the development of non-obese diabetic (NOD) mice, and a new strain of NOD/SCID mice was created by backcrossing SCID mice with NOD mice (Shultz et al., 1995). In these mice, the mature, function lymphocytes were absent, and lower levels of NK cells and cytokine production were present. Further studies were carried out by mating NOD/shi-SCID mice or *Rag2* mutation mice with interleukin-2 receptor gamma chain gene (*IL2rg*) mutation mice, which generated T, B, and NK cells combined deficiency mice, like NOG, NSG, and *Rag2/IL2*^-^*^/^*^-^ double knockout mice (Shultz et al., 2005; Belizário, 2009). These mouse have higher immunocompromised symptoms than the previously mentioned mice did due to the simultaneous absence of mature T-cells, B-cells, and NK cells as well as defective macrophage activity and reduced dendritic cell function (Ito et al., 2002; Shultz et al., 2007; McDaniel and Grisham, 2018). However, all of above-mentioned rodents were known as immunodeficient mice due to one or more immune response being impaired. As a result, these immunodeficient mice are advantageous because of their engraftment, infection control, and tumor control, and thus are a useful tool in biomedical research.

Clustered regularly interspaced short palindromic repeats (CRISPR) was a DNA loci in that it contained multiple, short, and direct repetitions of base sequences and could be found in bacteria and archaea (Jansen et al., 2002; Ishino et al., 2018). In the adjacent region of a CRISPR sequence, a conserved coding protein sequence was also identified, which was labeled as a CRISPR-associated (Cas) gene, and, correspondingly, its encoded protein was referred to as the Cas protein (Barrangou, 2015). Cas proteins form a large family that includes many subtypes; among these, the Cas9 protein originating from the bacterial type II CRISPR/Cas system is a programmable RNA-guided endonuclease that is capable of binding and cutting site-specific cleavage of double-stranded DNA (Mojica and Montoliu, 2016). The Cas9 enzyme recognizes the protospacer adjacent motif (PAM) sequence 5′-NGG-3′ and cleaves the DNA at 3 bp to 4 bp upstream of PAM guiding by tracrRNA and crRNA, the damage DNA is subsequently repaired using 2 main pathways: non-homologous end joining (NHEJ) and homology-directed repair (HDR). NHEJ always generates indel (insertion or deletion) mutations, while HDR occurs when the repaired template is presented (Hsu et al., 2014). The original biological function of CRISPR/Cas9 was an adaptive immune defense mechanism against phage for bacteria; the invaded DNA was recognized by CRISPR, after which Cas9 cleaved the exogenous DNA, leading the invading phage to become inactive (Sampson and Weiss, 2014). Since it represents a more convenient, rapid, and efficient way to introduce a mutation in a genome sequence, CRISPR/Cas9 is now known as a key novel genome engineering tool for replacing, deleting, or inserting base pairs into a DNA sequence, which could be used to construct genetically modified animals (Sander and Joung, 2014; Tschaharganeh et al., 2016).

Recombination activating gene 2 (*Rag2*), expressed in adult thymus (Wilson et al., 1994), is an immune-related molecule that is involved in the initiation of immunoglobulin V(D)J gene rearrangement and T-cell-receptor gene recombination during T- and B-cell development (Notarangelo et al., 2016). *Rag2* is essential to the generation of mature T- and B-lymphocytes; importantly, mutations of this gene in humans retards T- and B-cell development, resulting in SCID associated with autoimmune-like Omenn symptom occurrence (Corneo et al., 2001; Notarangelo et al., 2016). Separately, *IL2rg*, expressed in thymus and spleen (Cao et al., 1993), is known as an immune regulator in cytokine secretion; in the growth and differentiation of T-cells, B-cells, and NK cells; and in maintaining the homeostasis of the immune system (Aliyari et al., 2015). Mutation of the *IL2rg* gene prompted a deficiency in functional NK cell and cytokine secretion reduction, including IL-2, IL-4, IL-7, IL-9, IL-15, and IL-21 (Puck et al., 1997). In the present study, we postulated the construct of SCID mice through a mutation in the *Rag2* and *IL2*rg genes using the CRISPR/Cas9 gene editing tool, sought to determine the biological characteristics of the mutated mice by investigation the immune response against the 85B antigen of *Mycobacterium tuberculosis*, and establishment a human tumor xenograft model *in vivo*.

## Materials and Methods

### Animals

BALB/c mice were obtained from the Laboratory Animal Center of Air Force Medical University. ICR mice, which used as recipient animal for transplanting microinjected zygotes, were purchased from Vita River Laboratory Animal Technology Co., Ltd., (Beijing, China). The mice were housed in a temperature- and climate-controlled specific pathogen free facility with a 12-h light/dark schedule. Body weight and the intake of food and water were calculated per week. All mouse experiments were approved by the Institutional Animal Care and Use Committee of Air Force Medical University.

### Reagents and Plasmid

A MEGAshortscript^TM^ T7 high-yield transcription kit and MEGAclear^TM^ kit were provided by Thermo Fisher Scientific (Waltham, MA, United States). Cas9 messenger RNA (mRNA) and protein were purchased from Biomics biotechnologies (Nantong, China) and New England Biolabs (Ipswich, MA, United States), respectively. Pregnant mare’s serum gonadotropin (PMSG) and human chorionic gonadotropin (hCG) were purchased from the Ningbo Second Hormone Factory (Ningbo, China). M2 medium was provided by Sigma-Aldrich (St. Louis, MO, United States), while a KSOM powdered media kit (cat: MR-020P-SF) was obtained from Millipore (Burlington, MA, United States). Mouse FITC-CD3, PE-NKp46, and APC-B220 antibodies were purchased from BioLegend (San Diego, CA, United States). LongAmp Taq DNA polymerase and *Bbs I* restriction enzyme was provided by New England Biolabs (Ipswich, MA, United States). A mouse tail genome extraction kit was sourced from Foregene Biological Technology Co., Ltd., (Chengdu, China). pX330 plasmid was purchased from Addgene. Interferon (IFN) γ, IL-2, and IL-10 cytokine enzyme-linked immunoassay (ELISA) detection kits were purchased from eBioscience (San Diego, CA, United States).

### Cell Culture

The brain glioma cell line U87 was purchased from the Type Culture Collection of the Chinese Academy of Sciences (Shanghai, China). Human primary gastric, renal, and bladder carcinoma cell-luciferase and Passage Burkitt’s lymphoma cell line Raji-luciferase were obtained from the Laboratory Animal Center of Air Force Medical University. Cells were incubated in high-glucose Dulbecco’s modified Eagle medium or Roswell Park Memorial Institute 1640 supplemented with 10% fetal bovine serum under a humidified atmosphere of 5% CO_2_ at 37°C.

### Preparation of Single-Guide RNA and Microinjection

For the purpose of single-guide RNA (sgRNA) transcription *in vitro*, 2 20 bp sgRNA sequences targeting *Rag2* exon3 (gene ID: 19374) and *IL2rg* exon1 (gene ID: 16186) were screened on the website of http://crispr.mit.edu and synthesized by TsingKe Biological Technology (Xi’an, China). After annealing, double-strand DNA was digested with *Bbs I* restriction enzyme and cloned into pX330 plasmid. Polymerase chain reaction (PCR) was performed to obtain a sgRNA sequence carrying T7 promoter and the 121 bp PCR product then was transcripted with the MEGAshortscript^TM^ T7 high-yield transcription kit according to the manufacture’s protocol and purified. Mice superovulation and microinjection were carried out according to a previous report (Esmail et al., 2016). Briefly, 20 μg of *Rag2*, 20 μg of *IL2rg* sgRNA mixture, and 10 μg of Cas9 mRNA were microinjected into the cytoplasm of collected fertilized eggs. After incubation for 24 h at 37°C, the 2-cell forms of the eggs were then transplanted to the ampulla of recipient pseudopregnancy ICR female mice.

### Single-Guide RNA *in vitro* Cleavage Efficiency Assay

PCR reaction was performed with *Rag2* and *IL2rg* specific primers to obtain substrate DNA. After purification, 1 μg substrate DNA was digested with 2 μg Cas9 protein, 200 ng sgRNA, and 2 μL of 10 × Cas9 buffer at 37°C for 1 h in 20 μL of reaction volume. Reaction products were run on 1.5% agarose gel to examine cleavage efficiency.

### Flow Cytometry

50 μL of peripheral blood was collected from the tail veins of homozygous mice. Samples were lysed with erythrocyte lysing solution and incubated for 30 min with 1:1,000-diluted FITC-CD3, PE-NKp46, and APC-220 antibodies in a dark place. Then, samples were analyzed by flow cytometry (Becton, Dickinson and Company, Franklin Lakes, NJ, United States) and data were analyzed with the FlowJo softwares (FlowJo LLC, Ashland, OR, United States).

### Real-Time Quantitative RT-PCR

Total RNA was extracted from spleen and/or thymus of homozygotes mice with TRIzol reagent (Invitrogen, Carlsbad, CA, United States) according to the manufacturer’s instructions. 500 ng total RNA was reverse-transcribed to cDNA and qPCR was performed using a SYBR Green PCR kit (TakaRa, Dalian, China). Each sample was run in triplicate in a final volume 25 μl reaction mix, which contained 1 μl cDNA template, 10 pmol of *Rag2* and *IL2rg* specific primers (Table 1), and 12.5 μl of SYBR Green solution. Assays were run using following procedures: 1 cycle of 95°C 30 s, followed by 40 cycles of 95°C for 20 s and 60°C for 30 s. Data was analyzed with the 2^-ΔΔCT^ method.

**Table 1:** Primer sets used for qPCR.

Name of genes	Forward primer	Reverse primer
Rag2	ATTCAACCAGGCTTCTCACTT	TGCAGGCTTCAGTTTGAGATG
IL2rg	AGAGCAAGCACCATGTTGAA	CATTCGCACTGGACATGAGG
Actin	GGAAATCGTGCGTGACATCA	AATAGTGATGACCTGGCCGT

### Tumor Xenograft Model

15 *Rag2/IL2rg*^-^*^/^*^-^ mice were divided into three group: (1) human primary tumor cells inoculation group; (2) Raji cells inoculation group; and (3) U87 cells inoculation group. The logarithmic growth phase of human primary gastric, renal, and bladder carcinoma cells were collected and 1 × 10^7^ cells/mouse were implanted subcutaneously in the flank site and bred for 3 weeks. Meanwhile, 1 × 10^7^ Raji cells were inoculated intravenously to replicate a hematopoietic model. For the glioma xenograft model, 1 × 10^7^ U87 cells were stereotaxically injected into the precuneus, while the other mice were implanted subcutaneously in the flank site. 3 weeks later, all mice were euthanized and tumor formation was observed through skull anatomy in glioma xenograft mice, while the luciferase-labeled cell xenograft mice were visualized using the IVIS Lumina II imaging system (PerkinElmer, Waltham, MA, United States).

### Hematoxylin and Eosin Staining

Once the mice were euthanized, the thymus, spleen, and the xenograft tumor tissue samples were sectioned and fixed in 4% formaldehyde for 24 h, followed by dehydration with a series of ethanol solutions and subsequent embedding in paraffin. Then, 5-μm-thick sections were cut and stained with hematoxylin and eosin (H&E) according to protocol. The histopathological changes were examined under a light microscope (BX43; Olympus, Tokyo, Japan).

### Genotype Analysis

Genome DNA was extracted from the tail tip of 1-week-old mice and PCR reaction was performed with *Rag2* and *IL2rg* specific forward and reverse primers, respectively. After purification, PCR products were sequenced with Sanger sequencing and the results were analyzed with the SnapGene 3.1.1 software (GSL Biotech, Chicago, IL, United States).

### Immunization, Lymphocyte Proliferation, Antibody and Cytokine Assay

10 *Rag2/IL2rg*^-^*^/^*^-^ mice were divided into 2 group: (1) 85B antigen treated group; and (2) control group. Recombinant 85B antigen of *Mycobacterium tuberculosis* (MTB) was mixed with aluminum hydroxide adjuvant at a 1:1 ratio. Then, the mutated mice (experimental group) and WT BALB/c mice (control group) were inoculated 3 times intramuscularly with a 2-week interval by 50 μg of 85B antigen/adjuvant mixture at the hind leg. Following immunization, antibody, and lymphocyte proliferation assay were performed as done in a previous report (Zhang et al., 2010; Wang et al., 2014). Briefly, recombinant 85B antigen was coated and the titer of anti-85B specific antibody was carried out by ELISA assay. Meanwhile, lymphocytes were isolated from the spleen of immunized mice and simulated with recombinant 85B antigen (experimental well) or PPD (positive control), post which the supernatants were collected for cytokine measurement. Next, 20 μL MTS (Promega, Madison, MI, United States) was added in each well and incubated for another 4 h; optical density was measured under 490 nm; and the stimulation index (SI) was used to evaluate lymphocyte proliferation, as follows: SI = (A_490_ of stimulated wells – A_490_ of blank cells) / (A_490_ of negative wells – A_490_ of blank wells).

### Statistical Analysis

Data are expressed in the form of mean ± standard deviation. A Student’s *t* test and one-way analysis of variance were used for assessing significant differences among experimental groups using the Statistical Package for the Social Sciences software, version 17 (IBM Corp., Armonk, NY, United States). *p-*values of <0.05 or <0.01 were considered to be statistically significant.

## Results

### Construction of Rag2/IL2rg^-/-^ Gene Double-Knockout Mice With CRISPR/Cas9 System From BALB/c Strain

**FIGURE 1:**
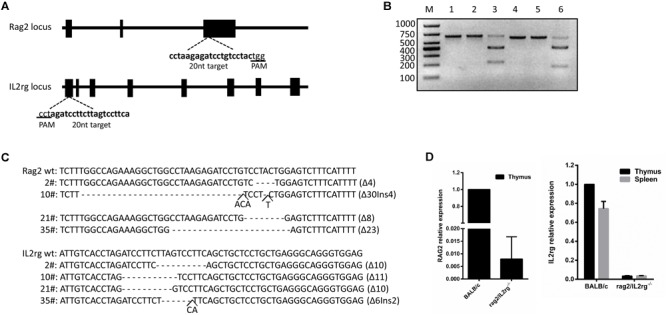
Construction of *Rag2*/*IL2rg*^-/-^ gene double-knockout mice with CRISPR/Cas9 system. Schema and sequence of sgRNAs targeted on Rag2 and IL2rg locus **(A)**. sgRNA cleavage efficiency. Genomic template was cleaved with or without Cas9 protein and sgRNA at 37°C for 1 h; products were then identified on 1.5% agarose gel. M, 1,000 bp marker; lane 1, Rag2 template; lane 2, Rag2 template + Cas9 protein; lane 3, Rag2 template + Cas9 protein + Rag2 sgRNA; lane 4, IL2rg template; lane 5, IL2rg template + Cas9 protein; and lane 6, IL2rg template + Cas9 protein + Rag2 sgRNA **(B)**. Genotype sequence of wild-type BALB/c and mutated mice in target region **(C)**. mRNA relative expression of *Rag2* and *IL2rg* in wild-type BALB/c and mutated mice **(D)**.

*Rag2* and *IL2rg* were involved in the development of T-, B-, and NK cells and the production of cytokines. The mutation of these genes retarded their development in the immature stage and contributed to a lack of both innate and adaptive immune response. Based on this, we planned to construct a SCID BALB/c mouse model targeting the *Rag2* and *IL2rg* genes simultaneously with a CRISPR/Cas9 gene editing tool. After screening on the http://crispr.mit.edu website, a pair of 20 bp oligonucleotides was selected as sgRNA targeting sequences from the *Rag2* exon3 sense strand and *IL2rg* exon1 anti-sense strand, respectively (Figure 1A). Subsequently, *in vitro* cleavage efficiency of the sgRNA assay demonstrated that *Rag2* and *IL2rg* sgRNA were endowed with stronger cutting activity for the target sequence, resulting in producing two obvious bands on 1.5% agarose gel (Figure 1B). Similarly, Sanger sequencing also suggested that the CRISPR/Cas9 system possessed higher gene editing efficiency *in vivo*. There were 40 pups born after transplantation, of which 20 pups (50%) and 18 pups (45%) showed induced indel mutation on the *Rag2* and *IL2rg* target sequences, respectively. Among these, 4 pups showed mutations simultaneously on both of these sequences, leading to small-fragment deletion or insertion (Figure 1C). Thus, 10# mouse was mated with wild-type female BALB/c mice to examine germline transmission. Three generations later, a similar genotype was observed in homozygote *Rag2*/*IL2rg*^-/-^ mice, suggesting indel mutations were stably inherited by offspring. *Rag2* and *IL2rg* expression was detected in adult thymus and/or spleen with specific primers (Table 1), data showed *Rag2* and IL2rg mRNA transcriptional level were decreased significantly (Figure 1D). Thus *Rag2/IL2rg*^-^*^/^*^-^ gene double-knockout mice were constructed based on BALB/c background.

### Rag2/IL2rg^-/-^ Double Knockout Alter the Number of Granulocytes, but Not Physiological Behavior of Mutated Mice

**FIGURE 2:**
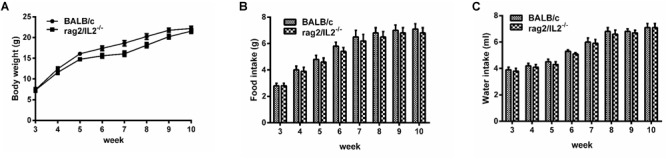
Physiological behavior of *Rag2*/*IL2rg*^-/-^ gene double-knockout mice. Average body weight of mutated mice measured per week **(A)**. Average food consumption of mutated mice measured per week **(B)**. Average water consumption of mutated mice measured per week **(C)**. Data are presented in the format of mean ± standard deviation of 3 independent experiments performed in triplicate (*^∗^p* ≤ 0.05, *^∗∗^p* ≤ 0.01, compared with controls).

Gene mutation might alter mouse phenotype, so we speculated as to whether *Rag2/IL2rg*^-^*^/^*^-^ double knockout influenced mutated mice’ normal behavior or not. Hematological parameters in routine blood test were assayed by biochemical analyzer. Data indicated that granulocyte counts for *Rag2*/*IL2rg*^-/-^ mice was decreased significantly, which resulting in increasing of percentage of neutrophils, monocytes, eosinophils and basophiles, while other hematological parameters unchanged (Table 2). There were no significant differences regarding body weight or food and water intake between mutated mice and wild-type mice (Figure 2A–C). Thus, *Rag2/Il2rg* mutation did not influence the physiological behaviors of mice.

**Table 2:** Comparison of Hematological parameters between Rag2/IL2rg^-/-^ and BALB/c mice.

Parameters	Rag2/IL2rg^-/-^	BALB/c	*P* values
HGB (g/dL)	16.76 ± 0.5	16.78 ± 0.4	0.956
RBC (×10^6^ cells/μl)	10.54 ± 0.31	10.69 ± 0.29	0.336
WBC (×10^3^ cells/μl)	1.61 ± 0.4	3.34 ± 0.37	0.000
PLT (×10^3^ cells/μl)	857.13 ± 73.21	730.38 ± 149.31	0.049
NEU (×10^3^ cells/μl)	0.99 ± 0.41	1.59 ± 0.34	0.007
LYM (×10^3^ cells/μl)	0.19 ± 0.07	1.41 ± 0.44	0.000
MONO (×10^3^ cells/μl)	0.09 ± 0.03	0.04 ± 0.02	0.000
EOS (×10^3^ cells/μl)	0.22 ± 0.14	0.11 ± 0.11	0.094
BASO (×10^3^ cells/μl)	0.01 ± 0.01	0 ± 0.01	0.159
NEU (%)	67.18 ± 7.3	47.83 ± 8.59	0.000
LYM (%)	11.71 ± 3.2	46.58 ± 7.28	0.000
MONO (%)	5.75 ± 0.93	1.06 ± 0.51	0.000
EOS (%)	14.65 ± 9.52	3.44 ± 3.09	0.012
BASO (%)	0.59 ± 0.61	0.14 ± 0.05	0.074

*p < 0.05 or <0.01 was considered statistically significant, compare with BALB/c mice.*

### Rag2/IL2rg^-/-^ Double Knockout Retarded Thymus and Spleen Development and Reduced the Number of Lymphocytes

**FIGURE 3:**
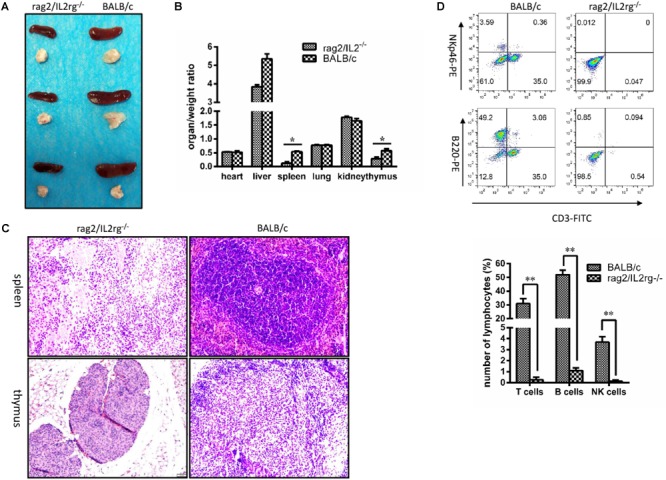
Changes of thymus, spleen tissue structure, and number of lymphocytes in *Rag2*/*IL2rg*^-/-^ gene double-knockout mice. Six-week mutated and wild-type BALB/c mice were sacrificed. Various organs were extracted, and the organ/weight ratio calculated **(A,B)**. Spleen and thymus tissues were sectioned and stained with the H&E method **(C)**. Peripheral blood was collected from tail veins and, after dilution, sample were stained with FITC-CD3, PE-NKp46, and APC-220 antibodies and detected by flow cytometry FCM **(D)**. Data are presented in the format of mean ± standard deviation of 3 independent experiments performed in triplicate (*^∗^p* ≤ 0.05, *^∗∗^p* ≤ 0.01, compared with controls).

Since *Rag2* and *IL2rg* genes were involved in immune-related tissue development, we attempted to investigate histopathological structure changes in thymus and spleen tissue. Notably, volume and organ weight ratio of thymus and spleen tissue were decreased significantly after mutation (Figure 3A,B). Thymus atrophy, spleen dysplasia, and lymphocyte reduction could be observed with H&E staining: thymus cells decreased and stromal cells increased in thymus tissue, cortical staining became lighter than the medulla, and the boundary between the cortex and medulla was blurred. Additionally, white pulp shrunk and red pulp expanded in the spleen tissue; the numbers of lymphocytes and hematopoietic and monocyte cells in white pulp were reduced; and the boundary between white pulp and red pulp was more unclear. However, the histopathologic structure of the spleen and thymus was normal in wild-type BALB/c mice (Figure 3C). Affected by this, the numbers of T-cells, B-cells, and NK cells in the peripheral blood were decreased significantly (Figure 3D).

### MTB Antigen 85B Could Not Stimulate Immune Response in Rag2/IL2rg^-/-^ Double-Knockout Mice

**FIGURE 4:**
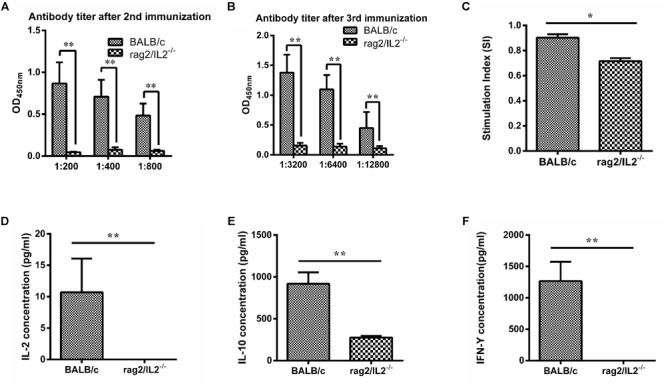
Immune response level induced by MTB 85B antigen. Recombinant 85B protein was immunized 3 times, the serum and spleen cells collected, and anti-85B specific antibody titer was detected by ELISA **(A,B)**. Spleen lymphocytes’ stimulation index (SI) was measured by MTS **(C)**. IL-2, IL-10, and IFN-γ concentrations in immunized serum were assayed by commercial ELISA kit **(D,E,F)**. Data are presented in the format of mean ± standard deviation of 3 independent experiments performed in triplicate (*^∗^p* ≤ 0.05, *^∗∗^p* ≤ 0.01, compared with controls).

*Mycobacterium tuberculosis* 85B protein was a prominent antigen designed to stimulate stronger cellular and humoral immune responses in inbred mice (Lu et al., 2018). To investigate the immune response induced by 85B antigen in *Rag2/IL2rg*^-^*^/^*^-^ double-knockout mice, recombinant 85B protein was immunized 3 times and the titer of anti-85B specific antibody was detected by ELISA. Higher-titer antibody could be induced in wild-type BALB/c mice, but not in *Rag2/IL2rg*^-^*^/^*^-^ double-knockout mice, even with an extension of immunization time (Figure 4A,B), and lymphocyte proliferation was inhibited because of lower SI (Figure 4C). Meanwhile, the expressions of IL-2, IL-10, and IFN-γ were decreased significantly (Figure 4D–F). Considering the above data, *Rag2/IL2rg* double knockout not only destructed the histopathological structure of thymus and spleen tissues but also attenuated the cellular and humoral immune responses in mutated mice, suggesting these mice presented the features of an immunocompromised animal.

### Rag2/IL2rg^-/-^ Double-Knockout Mice Were More Suitable for the Construction of Human Tumor Xenograft Model

**FIGURE 5:**
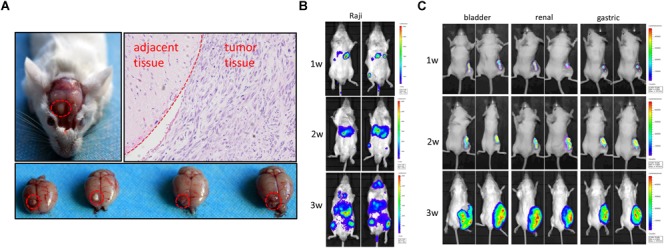
Replication of human tumor xenograft model in *Rag2*/*IL2rg*^-/-^ gene double-knockout mice. U87 cells were stereotaxically injected to the precuneus to replicate orthotopic transplantation of glioma. Tumor formation and histopathology structure were detected by anatomy and H&E staining **(A)**. Raji cells were injected intravenously into mutated mice and tumor formation was observed at different times by use of the IVIS Lumina II imaging system **(B)**. Primary gastric, renal, and bladder carcinoma cells were implanted subcutaneously in the flank site and bred for 3 weeks, and tumor formation was observed at different time by IVIS Lumina II imaging system **(C)**.

*Rag2/IL2rg*^-^*^/^*^-^ double knockout impelled SCID in mice; next, we attempted to transplant various human-tissue-derived primary and passage tumor cells into these mutated mice. To establish the orthotopic transplantation of glioma, U87 cells were inoculated intracerebrally into the mutated mice. 3 weeks later, tumor growth could be observed on parenchyma of the brain significantly. H&E staining showed tumor cell arrangement of dense, spindle cells. Notably, nuclear hyperchromatism and pathological mitosis were common in carcinoma, while abundant hemorrhage and necrosis were also observed in this region. Although tumor cell infiltration was displayed in the junction region, there was a clear boundary between carcinoma and paracancerous tissue, which was consistent with the pathological characteristics of glioma (Figure 5A). We also used lymphoma Raji cells to replicate a hematopoietic tumor model: as shown in Figure 5B, Raji cells penetrated the blood–brain barrier and distributed all over the body after intravenous injection, including in the brain (Figure 5B). Primary cultured cells of bladder cancer, renal cancer, and gastric cancer from clinical patients were transplanted subcutaneously, and the volume of xenotransplanted tumors was increased along with the time (Figure 5C). Taken together, our findings suggest *Rag2/IL2rg*^-^*^/^*^-^ double-knockout mice represent an ideal xenograft tumor model, as this mouse type showed compromised growth of various tissue-derived cancer cells and different inoculation methods.

## Discussion

In present study, severe combined immunodeficient mice were prepared by CRISPR/Cas9-mediated *Rag2* and *IL2rg* mutation. This mice was produced from clear genetic background of BALB/c inbred strain, with homozygosity as the main characteristic. Although severe combined immunodeficient mice could also be obtained by mating *Rag2* with *IL2rg* mutated mice (Belizário, 2009), but this mating might increase heterozygosity since *Rag2* and *IL2rg* mutated mice were from different strains. In addition, the biological characteristics of this immunodeficient mice were studied, the mice not only developed abnormal lymphatic organs, leading to number of immune cells decreased, but also could not induce immune response even stimulated with recombinant antigen, resulting in immune response defects. Interestingly, this attenuant immune response was more susceptible to compromising tumor xenotransplantation, which made this mice was more adapted to tumor xenograft model.

Gene mutation led to the production of an immunodeficiency animal. *Foxn1* gene mutation brought about a T-lymphocyte-mediated cellular immune response defect of hairless nude mice (Vaidya et al., 2016), *Xid* gene mutations produced a B-lymphocyte-mediated humoral immune response obstacle of CBA/N mice (Szymczak et al., 2013), and *beige* gene mutations also produced NK cell dysfunction of beige mice (Pflumio et al., 1989). At this point, these immunodeficiency animals showed only a single immune cell disorder. Gene mutations could additionally cause a variety of immune dysfunctions, with *prkdc* and *Rag2* spontaneous gene mutations having resulted in SCID mice. Although there were some similarities between these genes, *Rag2* was a vital kinase in the V(D)J recombination. T- and B-cell development was retarded when the *Rag2* gene was mutated because of V(D)J recombination (Carmona et al., 2016). At this moment, T-cell development was blocked at the immature CD3^-^CD4^-^CD8^-^CD25^+^ stage and B-cells were blocked at the B220^-^CD43^+^ IgM^-^progenitor B-cell stage (Riccetto et al., 2014; von Muenchow et al., 2017). The difference was that *prkdc* mutation led to immune leakiness in 20% of animals at 12 weeks of age, where immunoglobulin could be detected in the serum of *prkdc*-mutated mice (Danska et al., 1996; Katano et al., 2011). This interfered with the experimental results, but there was no immune leakiness in the *Rag2* gene mutation; that was the reason for why we selected the *Rag2* gene in this study.

Immunodeficient mice have been used to construct humanized animal models after the transplantation of human cells or tissues, so we attempted to explore the possibility of building a humanized animal after transplantation model involving human CD34^+^ hematopoietic stem cells and NK cells on *Rag2/IL2rg*^-/-^ mice. Unfortunately, transplanted CD34^+^ hematopoietic stem cells and NK cells abolished the ability of their survival and proliferation in mutated mice (data not shown), suggesting that mice with *Rag2/IL2r*^-/-^ gene mutation were not suitable for the construction of humanized mice. Several reasons might be considered as answers for this phenomenon: (1) sgRNAs of *Rag2/IL2rg* produced indel mutation in both of these genes, which induced small-fragment deletion, rather than the absence of large fragments. Although indel also could prompt frameshift mutation in *Rag2/IL2rg* genes, as compared with large-fragment absence, small-fragment mutation of indel seems less impactful on T- or B-cells. The utility of dual sgRNAs in follow-up studies to achieve the loss of large fragments of *Rag2*/*IL2rg* gene might be a better solution (Song et al., 2016). Additionally, (2) *prkdc*-mutated NOG or NSG mice were currently the best used humanized mice, although there was immune leakiness after *Prkdc* mutation. From the point of view of humanized mice construction, *prkdc* mutation was more suitable than *Rag2*, and (3) except for T-cells, B-cells, NK cells, neutrophils, macrophages, and cytokines could also cause a reaction of graft-versus-host disease, though the *Rag2/IL2rg* mutation reduced the number of neutrophils, lymphocytes in this study, residual granulocytes, macrophages, and cytokines might make it difficult for grafts to survive in mice. Although this immunodeficient mice were not suitable for humanized animal model, but it was a better tool for human tumor xenotransplantation, no matter orthotopic, hematopoietic, and xenotransplantatic tumor. Therefore, this immunodeficient mice was more propitious to application in tumor xenograft.

Except for applications in tumor research, immunodeficient mice could also be used in infection and immunity. Previous study demonstrated innate lymphoid cells were a critical role against *Clostridium difficile* infection based on data from *Rag1*^-^*^/^*^-^ single gene and *Rag2/IL2rg*^-^*^/^*^-^ double gene mutated mice (Abt et al., 2015). T, B cells were absent in *Rag1*^-^*^/^*^-^ and *Rag2/IL2rg*^-^*^/^*^-^ mice, however, innate lymphoid cells, like NK cells, Th17, and Th22 cells were normal in *Rag1*^-^*^/^*^-^ mice, but not in *Rag2/IL2rg*^-^*^/^*^-^ mice. Compared with *Rag1*^-^*^/^*^-^ mice, *Rag2/IL2rg*^-^*^/^*^-^ mice was succumbed to death after *C. difficile* infection owing to absence of NK cells, Th17, and Th22 cells. Thus innate lymphoid cells plays a protective role against *C. diificile* infection. Survival rate and cytokines expression, like IL-22, IL-17, and IFN-γ were assayed after challenge with *C. diificile* virulence strain. In present study, we evaluated the level of immune response, like antibodies titer, lymphocytes proliferation index, Th1 and Th2 cytokines after immunization with recombinant MTB 85B. Similarity, both mice abolished abilities of immune response, no matter immunization with virulence strain or recombinant MTB antigen. The difference was detection indexes, the immune properties of innate lymphoid cells was not investigated in this manuscript. Although we didn’t compare the difference of this two types mice, the immune properties should be similarity because both mice was mutated on same genes.

In summary, we have constructed a *Rag2/IL2* gene mutant mouse model using the CRISPR/Cas9 gene editing technology. The *Rag2/IL2* gene mutation did not affect the normal physiological behavior of mice, but the mutated mice displayed the typical characteristics of immunodeficiency. This mouse model could be used as a good animal model option in tumor research and other related fields.

## Author Contributions

HZ and ZC conceived the project. HZ designed the experiments. YoZ, PL, ZX, CS, YB, XS, YaZ, XW, LL, and XZ performed the experiments and prepared the manuscript. HZ and ZC supervised the study and contributed reagents and materials. All authors contributed to data analysis.

## Conflict of Interest Statement

The authors declare that the research was conducted in the absence of any commercial or financial relationships that could be construed as a potential conflict of interest.
